# 3D-printing inherently MRI-visible accessories in aiding MRI-guided biopsies

**DOI:** 10.1186/s41205-024-00227-w

**Published:** 2024-08-05

**Authors:** Yanlu Wang

**Affiliations:** 1 Department of Medical Radiation Physics and Nuclear Medicine, Karolinska University Hospital, Stockholm, Sweden; 2 Department of Oncology-Pathology, Karolinska Institute, Stockholm, Sweden

**Keywords:** Rapid prototyping, Medical devices, Phantoms, Biopsy, Radiology

## Abstract

**Background:**

3D printers have gained prominence in rapid prototyping and viable in creating dimensionally accurate objects that are both safe within a Magnetic Resonance Imaging (MRI) environment and visible in MRI scans. A challenge when making MRI-visible objects using 3D printing is that hard plastics are invisible in standard MRI scans, while fluids are not. So typically, a hollow object will be printed and filled with a liquid that will be visible in MRI scans. This poses an engineering challenge however since objects created using traditional Fused Deposition Modeling (FDM) 3D-printing techniques are prone to leakage. Digital Light Processing (DLP) is a relatively modern and affordable 3D-printing technique using UV-hardened resin, capable of creating objects that are inherently liquid-tight. When printing hollow parts using DLP printers, one typically requires adding drainage holes for uncured liquid resin to escape during the printing process. If this is not done liquid resin will remain inside the object, which in our application is the desired outcome.

**Purpose:**

We devised a method to produce an inherently MRI-visible accessory using DLP technology with low dimensional tolerance to facilitate MRI-guided breast biopsies.

**Methods:**

By hollowing out the object without adding drainage holes and tuning printing parameters such as z-lift distance to retain as much uncured liquid resin inside as possible through surface tension, objects that are inherently visible in MRI scans can be created without further post-processing treatment.

**Results:**

Objects created through our method are simple and inexpensive to recreate, have minimal manufacturing steps, and are shown to be dimensionally exact and inherently MRI visible to be directly used in various applications without further treatment.

**Conclusion:**

Our proposed method of manufacturing objects that are inherently both MRI safe, and MRI visible. The proposed process is simple and does not require additional materials and tools beyond a DLP 3D-printer. With only an inexpensive DLP 3D-printer kit and basic cleaning and sanitation materials found in the hospital, we have demonstrated the viability of our process by successfully creating an object containing fine structures with low spatial tolerances used for MRI-guided breast biopsies.

**Supplementary Information:**

The online version contains supplementary material available at 10.1186/s41205-024-00227-w.

## Introduction

In recent years, the widespread availability of cost-effective 3D-printers has revolutionized manufacturing and prototyping [1–4]. In the field of biomedical engineering, 3D-printing has been used for a variety of applications from creating bone models for precision medicine, to manufacturing tablet casings for medication [5, 6]. 3D-printing phantoms for radiology equipment is another useful application since individual phantoms are often designed with specific purposes and are costly. Here we present a novel and relatively easy method of 3D-printing objects that are natively visible in typical Magnetic Resonance Imaging (MRI) scans with DLP 3D-printers using an unorthodox printing technique.

### 3D-printing MRI phantoms

Hard plastics are generally invisible in typical MRI scans, hence 3D-printed MRI phantoms must be designed and printed hollow to contain some form of liquid, which are visible in typical MRI scans. It is challenging to create liquid-tight objects using Fused Deposition Modeling (FDM) 3D-printing. In FDM printing, an object is formed layer by layer using melted plastic. Micro-holes may arise between the individual layers of 3D-printed material, from which liquid may slowly seep through [7]. While it is possible to create liquid tight objects using FDM techniques, the process is not trivial, and success is not guaranteed. Fine-tuning printing parameters through trial and error for each individual 3D-printer may produce satisfactory results [8]. However, spatial accuracy is often sacrificed for structural soundness, which is not always desirable. Elaborate post-processing treatment of an object may also be applied, but additional materials and equipment may be necessary [9]. Furthermore, FDM 3D-printers lack the ability to create finely detailed parts.

### Digital light processing

Another type of 3D-printing technique which utilizes UV-hardened resin plastics has gained more prominence as costs decrease. Stereolithography (SLA) printers are typically costly to produce as they generally use UV-lasers to cure resin. More recently, Digital Light Processing (DLP) has been developed as an alternative to SLA printers with inherently lower cost since in DLP printers, UV-lasers are replaced by LCD panels and UV backlights. At the time of writing there is a wide selection of DLP 3D-printers which are priced in the consumer electronics range.

The process of object formation using UV-hardened resin fundamentally differs from FDM printing techniques. First, liquid UV-hardened resin is poured into a vat with a transparent film bottom. The vat is placed on top of a UV light source with LCD panel. During printing, a metal build plate is dropped into the vat, leaving only a thin layer between the bottom of the vat and the build plate. A pattern is shown through the LCD panel, exposing UV light to the thin layer or liquid resin, and curing it. The cured resin sticks onto the build plate and is then lifted from the bottom of the vat. After some time, the build plate lowers an appropriate amount again, leaving only a thin layer of space between the object and the bottom of the vat and the process iterates.

DLP 3D-printers can produce dimensionally accurate objects with a high resolution and good surface finish [10, 11]. The technique has been previously used to create phantoms for MRI, and other medical imaging modalities [4, 12–14]. DLP’s ability to recreate details has been shown to be suitable in printing dental models demanding a high degree of detail and surface finish [15, 16]. Objects created by curing resin in such fashion is also inherently liquid tight [17, 18]. To save on material costs, objects are typically hollowed out, leaving only the outer shell and perhaps some structural supports in the middle. Unhardened resin may get trapped inside during the printing process; hence drainage holes are inserted on the part towards the bottom of the build plate for the liquid resin to drain out into the vat during printing. However, if this is not done, liquid resin will remain trapped inside, which will be visible in MRI scans. We take advantage of this phenomenon, together with DLP’s high dimensional accuracy and ability to recreate fine details, to create objects that are inherently visible in MRI scans with low spatial tolerances efficiently at low cost.

### MRI-guided biopsy

One such application for this approach is to manufacture an MRI-visible grid system for MRI-guided breast biopsies. During MRI-guided biopsies, MRI scans of a patient’s breasts are acquired with a dedicated MR breast coil together with a spatial localization system towards the side of the breast of interest. MRI scans are of the breasts used to locate the region of interest for tissue extraction, while the localization system is then used to guide the biopsy needle toward the region of interest. It is therefore crucial that the MRI scans and the localization system are registered such that spatial localization in the MRI scans translate easily and accurately to physical space. This localization system is typically in the form of a grid made of hard plastic. Since the grid itself is invisible in the MRI scans, the grid must be pressed tight to make an imprint on the skin. This is not always possible as moving the grid on a rail system has limits, and since breast tissue is not infinitely deformable, the corresponding location of a target region might not be possible to obtain imprints on.

This poses a challenge when trying to correlate the region of interest for the biopsy with the spatial guides during the procedure, which may result in physicians cancelling the procedure due to lack of confidence in targeting or access to target [19, 20]. This is not ideal since MRI-guided biopsies comparatively complicated and expensive, and hence only requested when other modalities risk being inaccurate [21, 22].

### Purpose

Here we design, manufacture, and evaluate the feasibility of using DLP 3D-printing technology to produce an equivalent localization grid for MRI-guided biopsies that is inherently visible in MRI scans to facilitate the localization process between a target region and spatial guides for the biopsy entry point.

## Materials and methods

### Funding

This work was supported by the Swedish General Medical Fund under Grant FOUI-973889.

### Part design

**Fig. 1:**
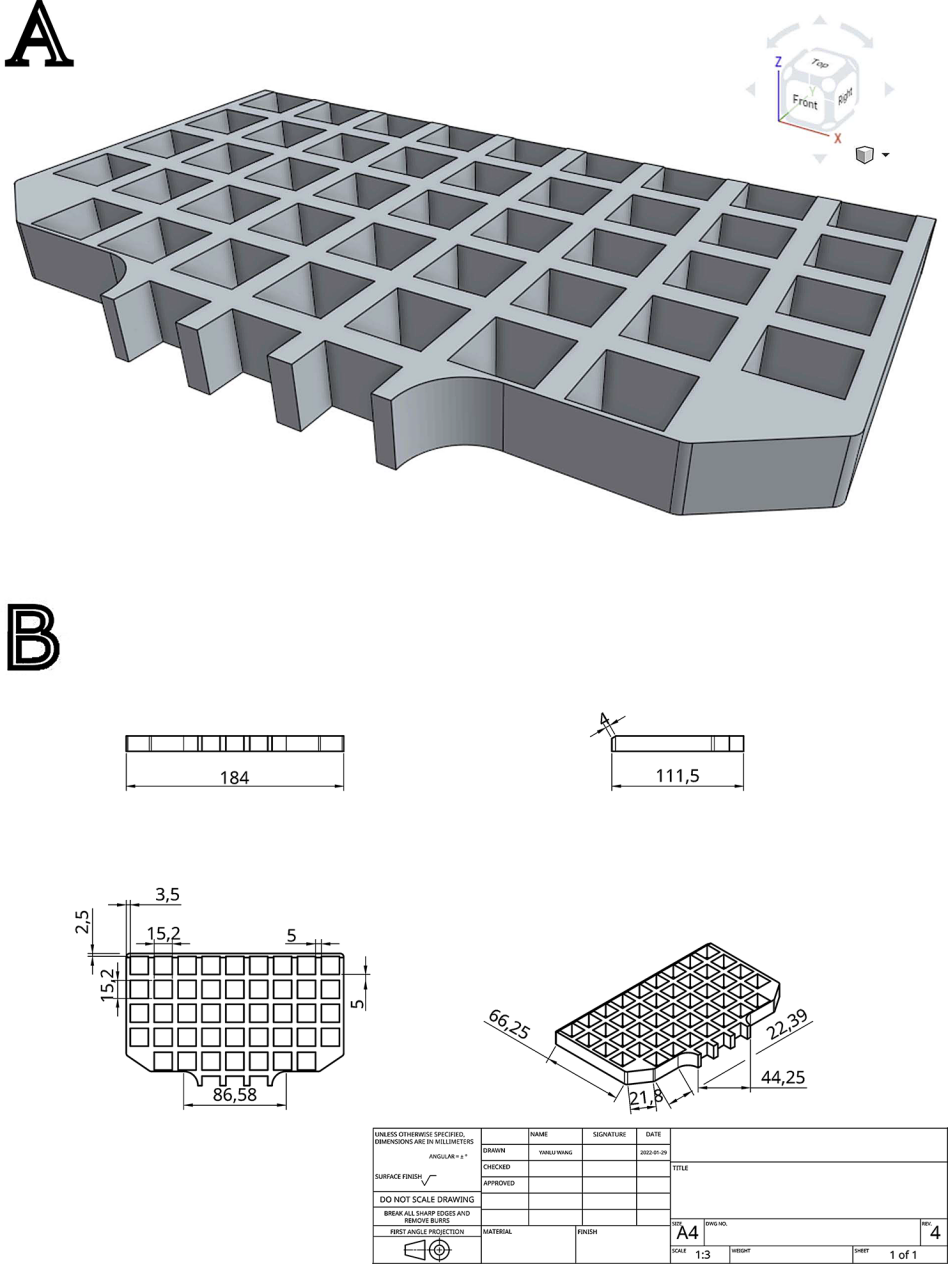
CAD drawing of the grid insert. The object is designed to fit inside the existing MRI-guided biopsy grid and to be printed hollowed out with a 1.4 mm thick wall. Since no drainage holes exist on the part, uncured liquid resin will be retained inside the part which is visible in the MRI scans. It is designed such that the liquid retained inside has the exact same dimensions as the original grid used for biopsy

Using 3D CAD software (Onshape, www.onshape.com), a model of the MRI-guided biopsy grid with the exact grid dimensions was created. The model is designed to fit inside the existing grid as an insert. The model then expanded 1.4 mm in all directions to act as an outer hardened shell to contain liquid resin (Fig. 1).

### Slicing

**Fig. 2:**
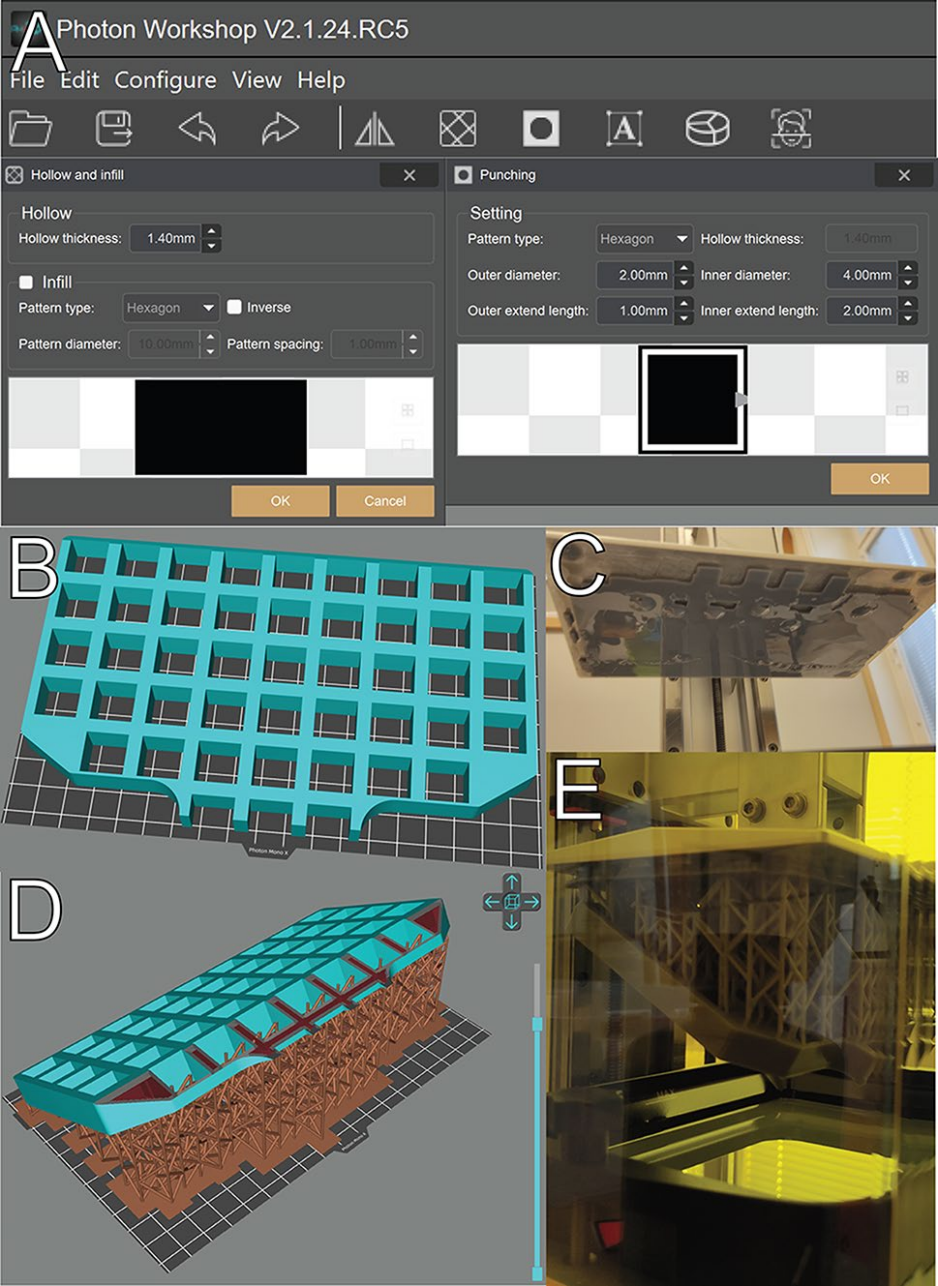
**A**: Slicer features: hollowing and drainage hole punching, are manipulated to retain liquid inside the part rather than draining it to save costs. **B**: Intuitive printing setup for this part with large, flat surfaces, this however causes layer adhesion issues and cause the print to fail easily (**C**). **D**: More robust printing layout with the part tilted at an angle (typically 30–60 degrees) and lifted slightly above the build platform with generated supports to hold up the part. This is more robust to printing failures (**E**)

Slicer software is used to convert a 3D-model into a format which 3D-printers operate on. Anycubic’s own proprietary slicer software Photon Workshop (V.2.1.24) was used since it is guaranteed to be compatible with our 3D-printer. Most slicer software for DLP printers have similar features to prepare a model for printing. These features include hollowing out a model to save on printing costs, and correspondingly, have a feature to insert drainage holes into the model at a given location for the unhardened resin within the model to leak out during/after printing. Here we do not wish for the unhardened resin to leak out, and hence will not insert any holes into the model, but we do wish to “hollow” out the model, creating a wall 1.4 mm thick (Fig. 2A).

Support generation is also another crucial step in preparing the design for 3D-printing which is also typically handled by the slicer software. In the final printing protocol we established, the model was placed slightly at an angle (30 degrees), and above (7 mm) the build plate, as this is the most robust way of printing the part (see Results). Photon Workshop’s automatic support generator was used to generate necessary support to build the part as specified (Fig. 1D).

Detailed slicer parameters including support generation parameters for the final object are found in (Table 1).

**Table 1:** Slicing, automatic support generation, and printing parameters

Setting Group	Parameter Label	Parameter Value
Slice Settings	Layer Thickness (mm)	0.05
	Expose Time (s)	10
	Off Time (Between exposure)	0.5
	Bottom Exposure Time	40
	Bottom Layers	10
	Z Lift Distance (mm)	4
	Z Lift Speed (mm/s)	2
	Z Retract Speed (mm/s)	3
	Anti-alias	1
Support Shape Settings		
Top	Contact Shape	Default
	Contact Depth (mm)	0.4
	Contact Diameter	0.8
	Shape	Cone
	Diameter (mm)	1.2
	Length (mm)	2.0
	Angle (degrees)	72
Mid	Shape	Cylinder
	Diameter (mm)	1.2
Bottom	Platform Touch Shape	Skate
	Touch Diameter (mm)	12.0
	Thickness (mm)	1.00
	Contact Shape	Default
	Contact Diameter (mm)	0.60
	Contact Depth (mm)	0.20
Raft	Shape	Default
	Raft Thickness (mm)	2.70
	Raft Inner Thickness (mm)	1.00
	Raft Angle	45
Support Generation Settings		
	Hollow Mesh	No
	Type	Vertical
	AutoSupportAngle	70
	Support Density (%)	70
	Support Min Length (mm)	3.00

### 3D-printing

A Photon Mono (Anycubic, https://www.anycubic.com/) was used to perform 3D-printing. A black tough (“ABS-like”) resin (Primacreator, article number 24,592) was used for the final object. Different resin types were also used during testing, such as “standard” resins in different colors (grey PV-RESIN-B405-0500-N and transparent PV-RESIN-B405-0500-CL) and water washable resin (PV-Resin-B405-1000-SK). The curing times are slightly different for the different types and colors of resin, so depending on the color of the filament, the curing times for each layer (and “first layers”) need be adjusted accordingly. For the print to be filled with liquid resin during printing, lift height after each layer was adjusted such that the part never lifts above the level of the liquid resin still in the vat. This may require manually filling the vat with fresh liquid resin in the middle of the printing process.

3D-printing is not an exact science and requires iterative improvement to achieve the desired results. Multiple prototypes were manufactured with various changes in design, printing parameters, and resins to achieve a robust printing process. The total print time depends on various printing parameters. Using parameters used for the final object (Table I), the total print time is approximately 6 h and 14 min.

### Post-production

Residue liquid resin remains on the surface after the print is completed, so the part still needs to be handled with caution (Fig. 2E). The object was removed from the print bed and the supports removed by hand in an isopropanol solution (40%) bath while wearing protective equipment. It was then further cured outdoors under the sun (summertime) for approximately 15–20 min. When sunlight is not easily accessible, UV light can also be used, including dedicated “washing and curing” equipment offered by different DLP 3D-printer manufacturers.

### MRI scanning

**Fig. 3:**
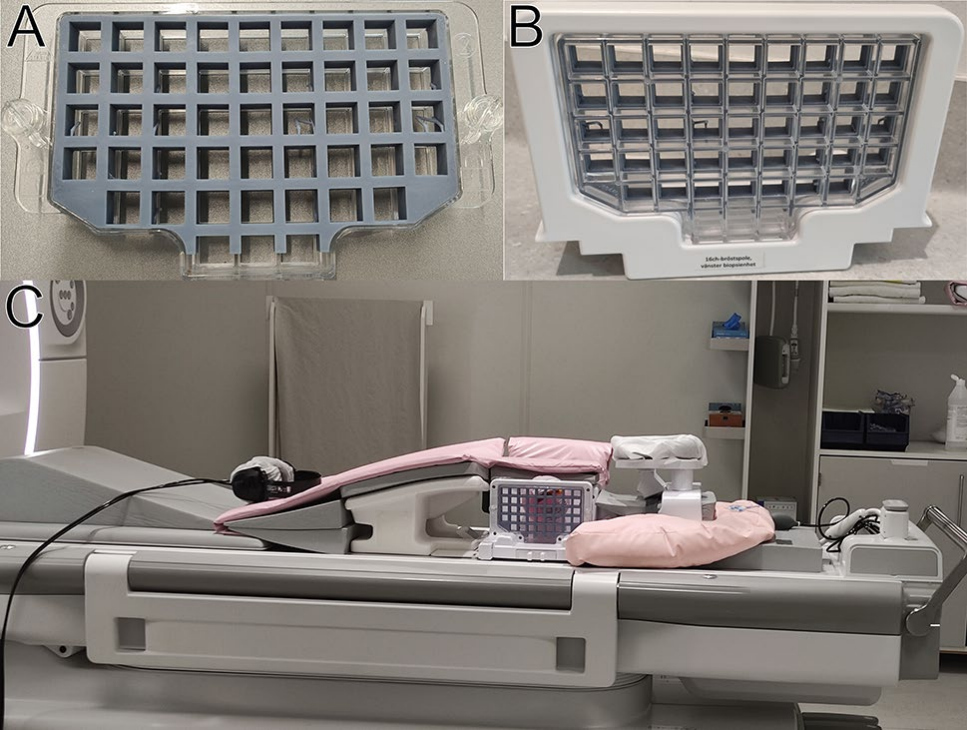
**A**: The finished part inserts into existing MRI-guided biopsy grid. **B**: the part on the grid in its retainer. **C**: The entire MRI-guided biopsy setup on the scanner bed

The finished part was then fitted onto the existing grid (Fig. 3A, B) and scanned with breast phantoms to assess visibility of the grid in MRI scans. Test scanning was performed on a GE Signa Premiere 3T MRI Scanner (Milwaukee, USA) with a 16-channel breast coil capable of MRI-guided biopsy procedures (NeoCoil), with one lateral side of the coil replaced with the grid setup as standard MRI-guided biopsy protocol (Fig. 3C). Both T1- and T2-weighted MRI sequences from our MRI biopsy protocol used at Karolinska University Hospital were used to assess grid visibility in typical MRI scans.

## Results

Since the designed part has many large flat surfaces, one would intuitively use one of such faces as the surface to adhere to the print surface (Fig. 2B). Experience shows that this makes the printing process more prone to failure (Fig. 2C) and the printing process is more robust when printing large flat surfaces at an angle (such that large surfaces are not printed at any single layer).

**Fig. 4:**
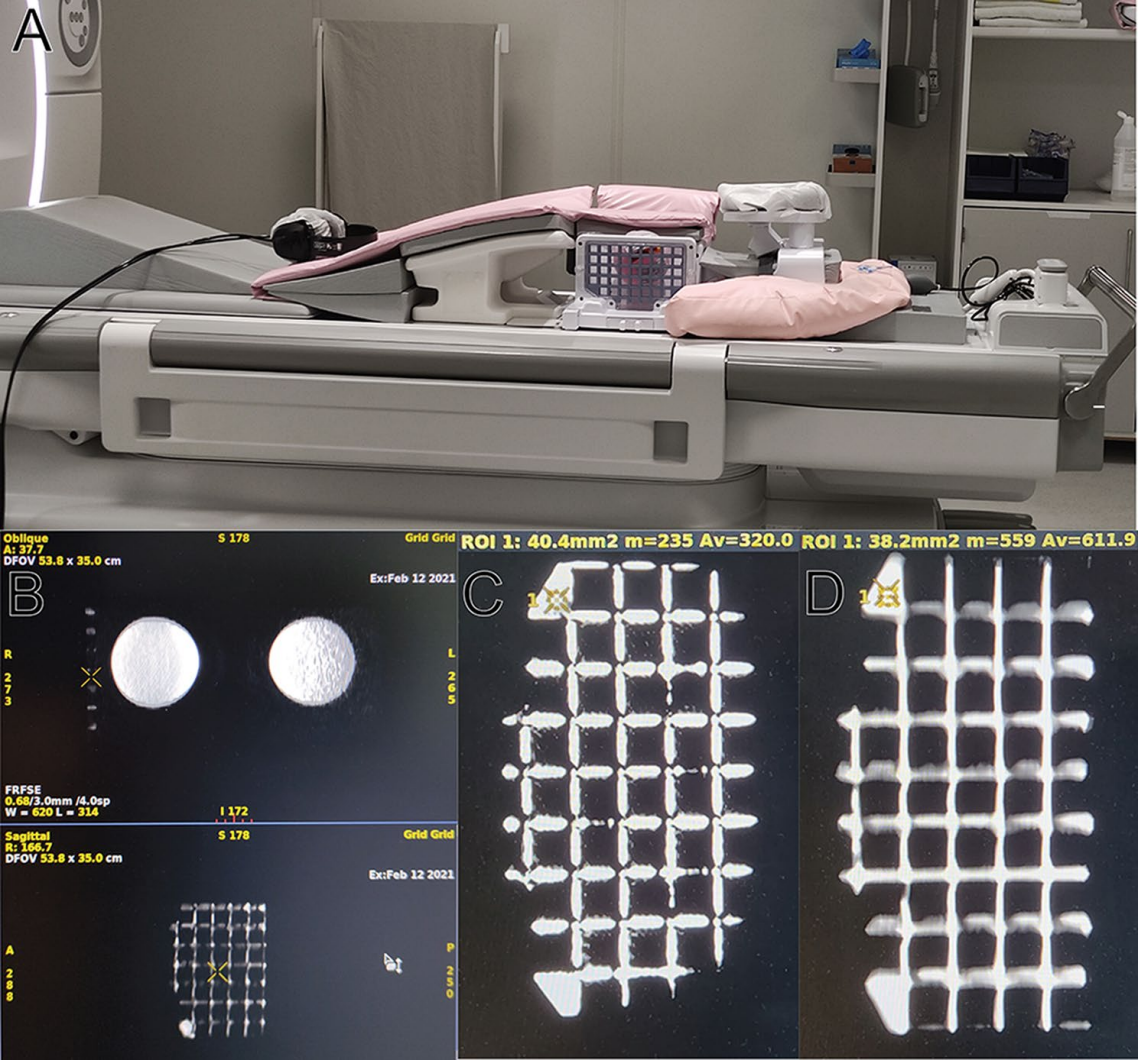
**A**: Even with MRI breast phantoms which are extremely hyperintense in MRI scans, the grid insert is clearly visible in the scans. **B**: Grid insert in T1-weighted MRI scan with ROI statistics. **C**: Grid insert in T2-weighted MRI scans with corresponding ROI statistics

The finished part should look and feel dry to the touch before handling using bare hands. The UV hardened outer shell is capable of fully enclosing liquid resin inside without leakage unless cracks appear on the outer shell (see Discussion). This is visible for the prototype manufactured in transparent clear resin, made specially for investigating the degree of liquid resin inside the part (see supplementary materials). The liquid inside the produced grid object is shown to be clearly hyperintense on both T1 and T2-weighted MRI scans (Fig. 4).

**Fig. 5:**
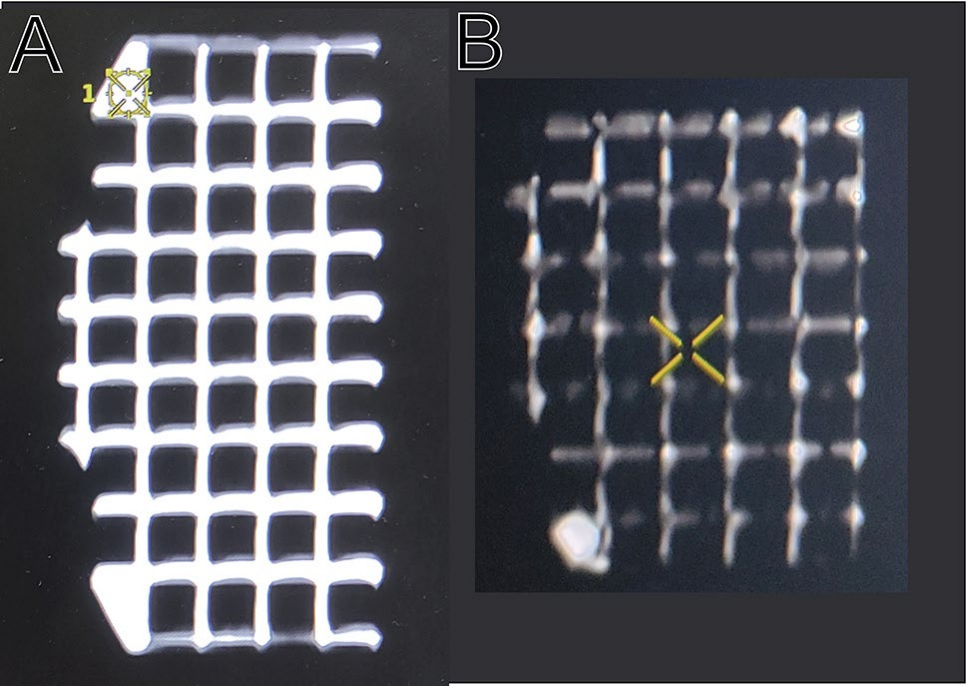
Differences in visibility in MRI scans between a grid insert prototype fully filled with liquid (**A**) and one which is only partially filled with liquid (**B**)

Tuning of printing parameters was needed to produce a filled object. An important parameter is the “Z-lift” length: It should be adjusted such that the lower edge of the unfinished print never lifts above the level of liquid resin remaining in the vat. This maximizes surface tension and will aid in retaining liquid resin inside the part during print through vacuum suspension. Depending on size and shape of the vat, manually filling the vat with fresh liquid resin during print might be necessary. Figure 5 shows the difference between a grid fully filled with liquid resin versus one that is only partially filled with resin due to suboptimal printing parameters.

## Discussion

Some downsides of the techniques are not only derived from the inherent downsides of the 3D-printing technique, but also from our unusual application of the technology. Any object produced in this manner must not only be 3D-printable in general, but the design must also allow for the sections visible in MRI scans to be fully enclosed by a hardened outer shell which lays additional restrictions during design.

The wall thickness of 1.4 mm presented in this study can be further optimized depending on materials used and individual use-case. From this study, our experience indicates that 1.4 mm outer walls can withstand normal handling, even for standard resins. Initially, 1.4 mm wall thickness was derived from the common 0.4 mm nozzles size from FDM printers and our experience that outer walls created only 2 passes of the 0.4 mm nozzle (0.8 mm) did not provide adequate structural integrity for small and delicate objects, while having 3 passes (1.2 mm) fared considerably better for most cases. This is however not directly translatable to DLP printing as materials and printing techniques differ considerably. For DLP printing, one is not practically restricted to have multiples of nozzle size as wall thickness to produce robust results, the wall thickness has been increased slightly from 1.2 to 1.4 mm to preemptively account for the more brittle material of standard resins used for DLP printing. However, this proved insufficient for standard resins to avoid cracking.

Since this method of production replies on liquid retention, the method of printing large objects in parts and then assemble them in post-processing in applicable in this case, hence the overall size of the object is restricted to the build volume the 3D-printer. Depending on the model of the 3D-printer, this may be a considerable restriction. Although more expensive 3D-printers capable of producing larger parts do exist, they are also considerably more expensive to acquire and run. In addition, filling a part with liquid resin adds significant production costs to the part compared to draining it.

**Fig. 6:**
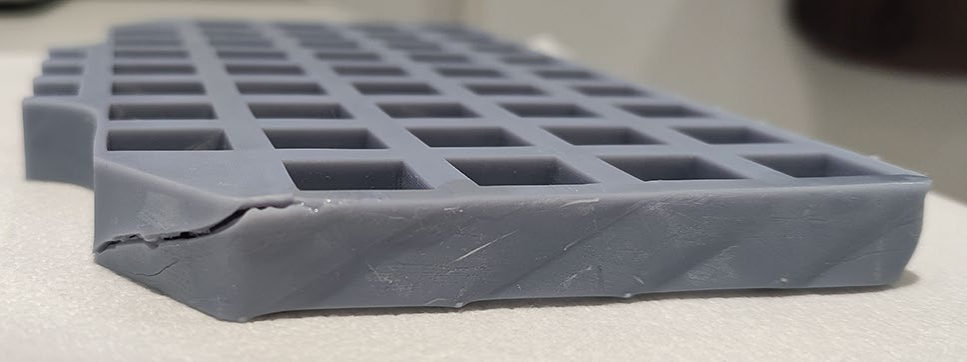
Using typical standard UV-hardened resin for DLP 3D-printers, cracks and fractures may arise on the outer cured shell of the part, which expands in time and may eventually cause leakages. Hence, “tough”, or “ABS”-like resin, or engineering resin may be preferred. Parts created by “tough” resins appear to be susceptible to cracks and fractures

Due to the brittle nature of the conventional UV-hardened resin, it would not be suitable to print such parts using the material. Fractures eventually appear on parts printing using “standard” resins (Fig. 6) after some time, ranging from a couple of days to a year. The fractures eventually expand and deepen resulting in liquid resin leakages. The exact cause of this is currently unknown and is yet to be investigated. A tougher material such as “ABS”-like, or engineering resin, which are both harder, and more malleable in their cured state is preferred since parts printed using these resins has yet to show fractures in their outer shells, even after more than a year from initial printing. The extent to which the walls can be thinned out without cracking for “tough” resins has yet to be explored.

This part is intended to be used multiple times, though the lifespan of parts made with the proposed method is still yet to be determined. As the liquid resin will harden under UV-light, it is recommended that the parts are stored in a dark space with minimal/no UV light penetration. While transparent resins will cause more UV shine-through and hence will cure more quickly, darker opaque colored resins may retain liquid inside for longer. Systematic investigation of MRI visibility over time for different resin colors is yet to be done. But preliminary experiments show that independent of resin color, the produced parts will eventually fully harden, and as such no parts produced in this manner will be permanently MRI-visible.

As all parts intended for medical use. The part able to endure sterilization, or at least be disinfected, with surgical alcohol without losing structural integrity or deforming. At our site, the part is disinfected using surgical alcohol prior to usage during MRI-guided biopsy even though the part does not directly make direct contact with the patient’s skin at any time during the procedure and cleaned using isopropanol after the procedure prior to storage. Excessive use of cleaning agents on the surface of the part may make the thin walls more brille and prone to breakage. Liquid resin contains irritants that are harmful to both humans and the environment. Even though the part does not make direct contact with the patient, there is always the additional risk of a patient coming into direct contact with liquid resin through cracks or leakage of any kind. In future patient trials, care must be taken to inspect the part for leakages prior, and after, use during procedures. Since the part will be not subjected to any considerable stress during the procedure, it is unlikely to crack during the procedure. Considering the benefits of including the part in procedures, patient risk is low, but not negligible.

A typical approach to making MRI phantoms is to design a hollow part and then fill it with a liquid. A grid may be produced using DLP/SLA with multiple drainage holes to allow uncured resin to flow out and then sealed after filling with either water or oil. Similarly, a hollow object can be made with a well calibrated FDM printer that allows for robust production of liquid-tight objects. All methods will have the same effect in terms of visibility in MRI scans, but with additional cost in tools, materials, and production effort/time. Tuning an FDM printer to robustly produce liquid-tight and detailed parts is a time-consuming task and relies heavily on trial and error. Draining, washing, and curing the inside of a hollow SLA/DLP part is not a trivial task, and will typically require additional precision tools. While hollow parts filled with water or oil are arguably safer and longer lasting, the proposed method is easier, and is less time-consuming and doesn’t require additional tools during manufacturing and post-processing.

This production process can be used to efficiently manufacture various devices designed to be inherently visible in MRI scans, such as phantoms and markers. Due to the great spatial accuracy of the 3D printing technology, the produced parts will not only be inherently liquid tight, but also dimensionally accurate for quality assessment applications.

## Conclusion

Using relatively inexpensive and simple DLP 3D-printing techniques, we can construct dimensionally accurate parts that are inherently visible in typical MRI scans. The produced part is designed specifically to aid in spatial localization of tumors in MRI-guided biopsy procedures, greatly simplifying the procedure and enhancing confidence for personnel in the process.

### Electronic supplementary material

Below is the link to the electronic supplementary material.

Supplementary Material 1

## Data Availability

No datasets were generated or analysed during the current study.

## Declarations

This is a pilot study and currently not a part of any Clinical Trials.
